# Description of clinical pharmacists reported interventions to prevent adverse drug events among patients with cardiovascular disease in Qatar

**DOI:** 10.5339/qmj.2024.27

**Published:** 2024-08-27

**Authors:** Rawan Abouelhassan, Dina Abushanab, Moza Al Hail, Wessam El-Kassem, Palli Valappila Abdul Rouf, Rasha Kaddoura, Fatima AlMaraghi, Ahmed Mahfouz, Sumaya Alyafei, Daoud Al-Badriyeh

**Affiliations:** 1 Department of Research, Sidra Medicine, Doha, Qatar; 2 Department of Pharmacy, Hamad Bin Khalifa Medical City, Hamad Medical Corporation, Doha, Qatar; 3 Department of Pharmacy, Heart Hospital, Hamad Medical Corporation, Doha, Qatar; 4 College of Pharmacy, QU Health, Qatar University, Doha, Qatar *Email: daoud.a@qu.edu.qa

**Keywords:** pharmacy, intervention, medication, drug-related problems, patient safety

## Abstract

**Background:**

Unidentified drug-related problems (DRPs) can cause negative health and economic consequences if not addressed appropriately. The literature revealed that interventions conducted by clinical pharmacists can positively impact patient safety and treatment outcomes. The role of clinical pharmacists has been continually growing while posing significant improvements in the provision of healthcare.

**Objective:**

To describe clinical pharmacist interventions in hospitalized patients with cardiovascular disease (CVD) in Qatar.

**Methods:**

This is a retrospective analysis of clinical pharmacist interventions documented in an electronic patient medical database. Data were retrieved from three date ranges and comprised demographic information, interventions, medical wards, drug therapy, and medical disorders. Clinical pharmacist interventions were categorized using a standardized intervention reporting sheet from the medical database in the hospital.

**Results:**

A total of 845 interventions relating to 262 patients were included in this study. The study population consisted mainly of males (*n* = 158 [60%]) with an average age of 61 years (SD ± 13.05). The leading documented interventions were the addition of medication (*n* = 278 [32.9%]), medication discontinuation (*n* = 196 [23.2%]), and an increase in medication dosage (*n* = 155 [18.3%]). A similar trend was observed throughout all subcategories investigated in this study, that is, interventions according to age, ward, and gender. An exception to the common trend was demonstrated in the emergency department, where medication discontinuation was the most frequent intervention. The classes of medications with the highest frequency of reported interventions included cardiovascular medications, followed by endocrine and hormonal agents (*n* = 393 [46.51%] and *n* = 159 [18.7%], respectively).

**Conclusion:**

Interventions conducted by clinical pharmacists have proven to have a positive impact on patient safety in addressing and resolving DRPs. Healthcare systems may benefit from future efforts directed toward studies of a prospective nature while developing a unique indicator of the validity and precision of documented interventions.

## Introduction

Pharmaceutical care highlights the crucial importance of practicing safe and effective therapeutic patient care. Notably, patient safety is one of the most essential aspects of a healthcare system, as recognized by numerous international organizations.^1,2^ Efforts by healthcare professionals, primarily clinical pharmacists, have been dedicated to ensuring the safety of patient management while aiming to identify drug-related problems (DRPs), reverse medication errors, and avoid adverse drug reactions (ADRs) or adverse events (AEs).^3,4^ A DRP is defined by the Pharmaceutical Care Network Europe Association as an event or circumstance involving drug therapy that actually or potentially interferes with desired health outcomes.^5^ In addition, there is a wide array of DRPs that may be observed in healthcare systems, where the American Society of Health-System Pharmacists acknowledges eight types of DRPs, including ADRs, medication errors, drugs without indication, failure to receive medication, drug interactions, noncompliance, untreated indications, and drug poisoning.^6,7^

Furthermore, DRPs may escalate into serious health issues such as the possible increase in the length of hospital stay, intensive care unit (ICU) admissions, serious morbidity, economic burden, and, in certain cases, the increased risk of death.^8–13^

Within this context, previous literature has aimed to investigate the effectiveness of several approaches that may contribute to minimizing DRPs, such as clinical pharmacy services, automated systems, and specific forms of technology.^14,15^ Numerous studies conducted in different healthcare settings have observed several types of interventions conducted by pharmacists that were documented through medication reconciliation programs, quality improvement projects, and clinical pharmacist interventions. Particularly, the role of clinical pharmacists in conducting interventions was significantly highlighted, whereby they take immediate actions to address non-optimal prescribing behavior to avoid undesirable consequences, improve the quality of life, and minimize unnecessary health expenditure.^16–19^ Notably, key findings from previous literature have demonstrated the significant impact of pharmacist interventions on patient care and, subsequently, the overall beneficial effect on the culture of patient safety as well as the considerable prevention of possible complications that may have resulted from the unsolved DRPs.^20–22^

In recent years, various studies have been reported in the Middle East, analyzing the impact of clinical pharmacist services on healthcare systems. Studies conducted in countries including Saudi Arabia, the United Arab Emirates (UAE), and Oman have demonstrated the positive impact on clinical, economic, and humanistic outcomes while highlighting strategies used to address medication errors and DRP.^23–25^ In Qatar, prior studies have investigated the significance of the clinical pharmacist’s role in contributing to health outcomes, where two studies investigated the effect of clinical pharmacist interventions (CPIs) on addressing and resolving DRPs. These, however, were primarily reported in ambulatory care settings.^26,27^ Hence, to our knowledge, there have been no previous studies that aimed to investigate the benefits of CPIs on DRPs in hospitalized patients in Qatar. Therefore, this study aims to characterize and analyze clinical pharmacist interventions to minimize ADEs due to DRPs in hospitalized patients at the Heart Hospital in Qatar.

## Methods

### Ethical approval

The study was approved by the Medical Research Center, Hamad Medical Corporation (HMC), in 2019 (MRC-01-19-110).

### Study design and setting

A retrospective analysis of CPIs documented in an electronic patient medical database (Cerner) at the Heart Hospital (HH), Hamad Medical Corporation (HMC), Doha, Qatar. This hospital setting is known as the leading contributor to the secondary and tertiary healthcare provision of cardiology services, with 139 beds, in Qatar. The CPI is defined as “any action by a pharmacist that directly resulted in a change to patient management or therapy.^27^” The respective interventions are routinely documented in an electronically embedded intervention sheet found in Cerner.

### Sample population

CPIs conducted in any adult patient admitted to HH and reported in Cerner during the date periods between March 1^st^ and March 31^st^, 2018; between July 15^th^ and August 15^th^, 2018; and between January 1^st^ and January 31^st^, 2019 were considered eligible to be included in this study. Notably, only CPIs approved by physicians were included since clinical pharmacists merely place recommendations to conduct an intervention; a physician’s approval is required so that the intervention is executed and patient care is altered accordingly. The CPIs that were conducted by operational pharmacists were excluded. The frequency and types of CPIs were described according to several subgroups, including, age group, gender, class of medications, and medical wards. Age groups were classified as adult (less than 65 years of age) or elderly (65 years of age or older).

### Data extraction and synthesis

Demographic data relating to the patient population as well as the description of CPIs were extracted from a standardized CPI reporting system in Cerner and recorded in an electronic data collection sheet. The CPI categories and their subtypes were obtained from the standardized sheet, including (1) involved medication, (2) pharmacological class, (3) route of administration, (4) prescriber response, and (5) intervention type: appropriate therapy, contraindication/safety, dosing/administration, drug interaction, duplicate therapy, non-formulary, drug information, medication reconciliation, and incomplete prescription. When data is not available through the CPI reporting system (e.g., patient or medication-related information), the medical records for the respective patients would then have to be reviewed in order to obtain the data. Two members of the research team evaluated all the extracted intervention details.

### Data analysis

Data analysis was completed using IBM Statistical Package for Social Sciences (IBM SPSS Statistics, Version 24.0; IBM Corp., Armonk, NY). Continuous variables were presented as means ± standard deviation (SD), while categorical variables were displayed as frequencies and percentages.

## Results

### Description of study population

**Figure 1.**
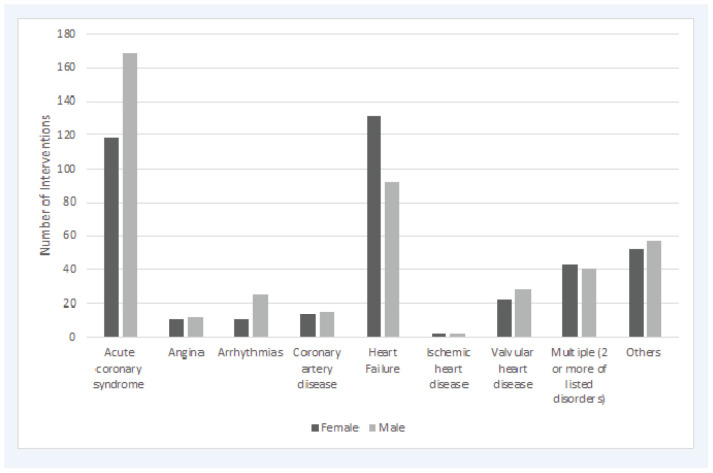
Frequency of pharmacist interventions according to active medical disorders.

A total of 262 patients in the HH were enrolled in this study with a total of 845 reported CPIs, taking place across 3 months within an 11-month period (March 2018 to January 2019). The majority of the patients were male (*n* = 158 [60%]) and the mean age of the study population was 61 years. As reported in Table 1, more than (*n* = 197 [75%]) of the patients were Arab and were admitted to the inpatient general cardiology unit. Furthermore, Figure 1 highlighted the most common active cardiovascular medical disorders for which CPIs were reported: acute coronary syndrome (ACS) (*n* = 287 [33.96%]) and heart failure (*n* = 223 [23.79%]). The most frequently occurring active medical disorder in the male population was ACS (*n* = 169 [59%]), while heart failure was the highest amongst the female population (*n* = 131 [58%]).

### Types of CPIs

CPIs were reported based on a classification of the types of interventions listed in Table 2. The most abundant CPI documented was “addition of another medication” (*n* = 278 [32.9%]), accompanied by “discontinuation of medication” and “increase in medication dose,” with (*n* = 196 [23.2%]) and (*n* = 155 [18.34]), respectively. The CPIs that were minimally reported included “decrease in medication duration” (*n* = 2 [0.24%]) as well as “increase in medication frequency” (*n* = 1 [0.12%]).

### Description of CPIs

**Figure 2.**
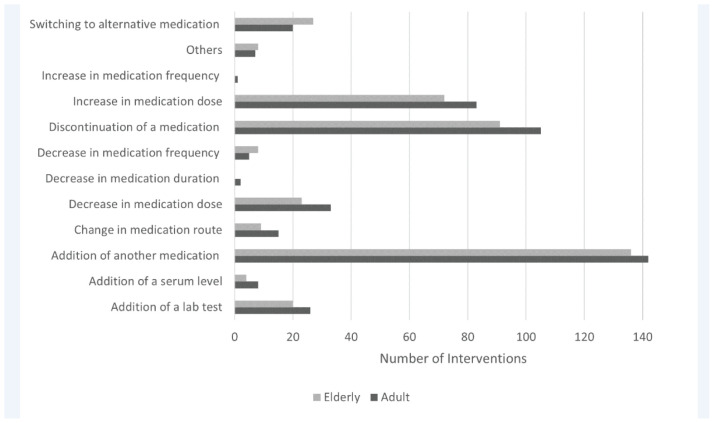
Types of interventions according to age group.

The majority of the documented CPIs were reported in March 2018 (*n* = 456 [54%]), while 282 (33.4%) CPIs took place between July and August 2018, and 107 (12.6%) CPIs were reported in January 2019 (Table 1). The occurrence of the various types of CPIs was investigated according to subcategories where “addition of another medication” was the most commonly reported amongst the adult and elderly (above 65 years of age) as well as the male and female subgroup populations (Figure 2 and Table 3, respectively). Particularly, this CPI was reported more frequently in the male population as compared to the female population (*n* = 145 [51.7%] vs. *n* = 133 [48.2%]), while it was almost equally present amongst the adult and elderly populations (*n* = 142 [51%] and *n* = 136 [49%]), respectively.

### Description of CPIs according to class of medication

**Figure 3.**
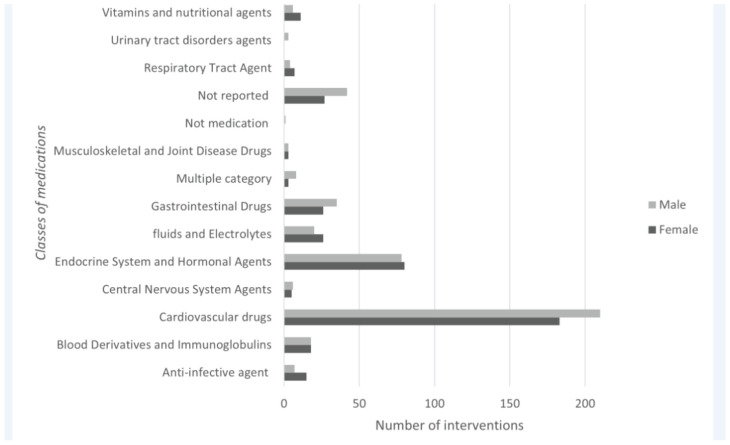
Classes of medications according to gender.

Out of the 845 CPIs analyzed in this study, 393 (46.5%) were related to cardiovascular medications, which comprised primarily antihypertensive drugs, anticoagulants, and antiplatelets, among others (Table 4). Specifically, from this class, the most frequently reported medications included bisoprolol (*n* = 23 [5.8%]), warfarin (*n* = 26 [6.6%]) and aspirin (*n* = 5 [2%]). Additionally, the majority of reported CPIs in relation to this class of medications were “addition of another medication,” followed by “discontinuation of a medication,” and “increase in medication dose,” at 14.3% (*n* = 56), 12.3% (*n* = 49), and 9.8% (*n* = 39), respectively. On the contrary, only one CPI suggested the addition of a serum level and a lab test relating to cardiovascular agents. The second most commonly observed class of medications was endocrine system and hormonal agents, which typically encompass antihyperlipidemic agents, antidiabetic agents, steroids, and thyroid agents, among many others. Precisely, from this class, insulin glargine (*n* = 37 [23.4%]) and atorvastatin (*n* = 34 [21.5%]) were the agents with the most documented CPIs. According to the commonly observed trend in this study, the highly reported CPI under this class of medications was “increase in medication dose” (*n* = 66 [16.8%]), followed by “addition of another medication” (*n* = 45 [11.5%]). Two other types of CPIs were also abundantly reported within this class of medications, including “switching to an alternative medication” and “discontinuation of a medication” (*n* = 16 [4.1%] and *n* = 17 [4.3%], respectively). With regards to the medication use per gender, cardiovascular drugs were the most common amongst the male population (*n* = 210 [24.9%]), while CPIs relating to endocrine agents were more common in females (*n* = 80 [9.5%]) (Figure 3). Both classes were more frequently present in CPIs conducted in the adult population as compared to the elderly population (Table 5).

### Description of CPIs according to hospital ward

#### General cardiology units

**Figure 4.**
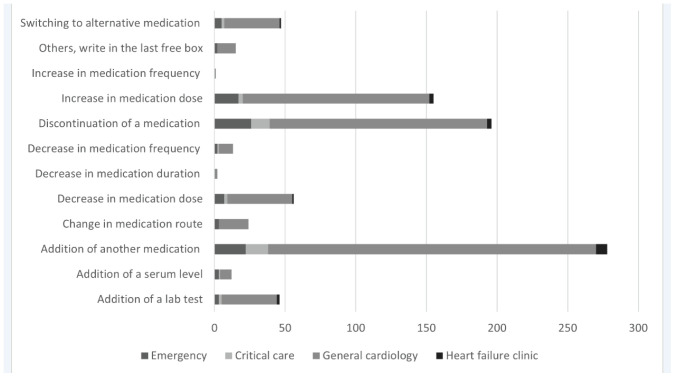
Types of interventions according to ward.

A total of 697 (82.5%) CPIs were documented for patients admitted to the general cardiology units. “Addition of another mediation,” “discontinuation of a medication,” and “increase in medication dose” were the three most commonly reported CPIs in this patient setting (*n* = 232 [33.3%], *n* = 154 [22.1%], and *n* = 132 [18.9%], respectively) (Figure 4). Similarly, “addition of another medication” was reported to be the highest amongst the adult population admitted to the general cardiology unit as compared to the elderly (*n* = 111 [29.1%] vs. *n* = 93 [15.9%]). This CPI was also commonly observed in the male population admitted to the general cardiology unit as compared to females (*n* = 81 [17%] vs. *n* = 73 [16.2%]). The majority of the CPIs in this unit were related to cardiovascular drugs (*n* = 317 [45.5%]) and endocrine system/hormonal agents (*n* = 141 [20.2%]), followed by gastrointestinal drugs (*n* = 50 [7%]) and fluids and electrolytes (*n* = 40 [5.7%]). The least occurring classes of medications were central nervous system agents and musculoskeletal and joint disease drugs (Table 6).

#### Critical care units

A total of 40 CPIs were conducted in critical care units. Most of the CPIs were reported for male and elderly patients (Tables 5 and 6). A similar trend of frequently occurring CPIs was demonstrated in the critical care unit as that observed with general cardiology (Figure 4). Only two CPIs were documented for each “addition of a lab test,” “decrease in medication dose,” and “switching to alternative medication,” while one intervention was reported for “addition of a serum level” and “decrease in medication frequency.” Table 6 demonstrates that the common classes of medications for which CPIs were documented in the critical care unit primarily include cardiovascular drugs and blood derivatives and immunoglobulins (*n* = 20 [50%] and *n* = 4 [10%]), respectively.

#### Emergency department (ED)

Out of the 845 CPIs observed in this study, 90 (10.7%) were documented in the ED. Unlike the trends observed in the previous general and critical care units, with “addition of another medication” being the most commonly reported type of CPI, the ED demonstrated that “discontinuation of a medication” was the most occurring CPI, followed by “addition of another medication” (Figure 4). As seen in all hospital settings, most CPIs were reported in male and elderly patient populations. With regards to CPIs under classes of medications, the same classes were frequently observed in both the general cardiology and ED units, where cardiovascular drugs were the highest, followed by endocrine and hormonal agents (Table 6).

#### Heart failure clinic

The clinical pharmacists practicing in the heart failure clinic documented a total of 18 CPIs. In this case, however, the majority were observed in female and elderly patient populations. The commonly occurring CPIs in this setting demonstrated a similar pattern to those observed in the critical and general cardiology settings, with “addition of another medication” being the highest amongst all the CPIs (n = 8 [44%]) (Figure 4). Similar to the critical care units, the most frequent classes of medications with reported CPIs were cardiovascular drugs and blood derivatives and immunoglobulins; n = 11 (61%) and n = 3 (17%), respectively (Table 6).

## Discussion

This is the first study of its kind to be conducted in a specialized cardiology hospital in Qatar, aiming to describe and analyze interventions placed by clinical pharmacists. The most prominent finding that was observed in this study was that the addition of another medication was the most frequently occurring CPI amongst the total of analyzed CPIs, followed by discontinuation of a medication. This is inconsistent with findings that were observed in studies conducted in the United Kingdom as well as the Kingdom of Saudi Arabia, where the most commonly reported CPIs in such hospital settings were relating to the dosing of medications (i.e., incorrect dose, increase dose, or decrease dose).^28–30^ It is worth mentioning that the majority of previously published studies did not consider the addition of medications as part of the categories of interventions.^18,21^,^23^ While several studies demonstrated the occurrence of interventions relating to the alteration of drug therapy, such studies were conducted in outpatient settings, with examples demonstrated in the Kingdom of Saudi Arabia, the United Arab Emirates, the ambulatory care setting in Qatar, and the community pharmacy setting in the United Kingdom.^19,25^,^27,30^ Such therapy-altering interventions significantly highlighted the role of the clinical pharmacist since pharmacists are known to significantly contribute to the identification of medical conditions that have not been receiving adequate treatment or conditions that have not been diagnosed previously and, hence, require necessary treatment as recommended by the clinical pharmacists.^29,30^ Furthermore, the second most reported CPI in this study was related to the discontinuation of unnecessary medications, which was considered consistent with findings reported in numerous studies that investigated CPIs in several patient settings.^25,27^,^28^ Considering that pharmacists possess specialized knowledge of medications and their indications, such results were highly anticipated and were consistent with the current study. Moreover, given the nature of the heart hospital setting investigated in this study, it was expected that the majority of the medical conditions would be of a cardiovascular nature. Hence, the most commonly witnessed class of medications for which CPIs were documented was cardiovascular medications. This did not directly align with findings from other studies since this was the first study to outline CPIs in a specialized heart hospital setting. Nevertheless, previous studies demonstrated that cardiovascular agents were frequently the second most recorded medications for which CPIs were conducted, seeing that cardiovascular disease is one of the leading medical conditions in the world, with the most common medications being anti-infective agents.^31,32^ Specifically, from this pharmacological class, the CPIs were most frequently associated with bisoprolol, warfarin, and aspirin, where the majority of CPIs reported under this class were “addition of another medication,” followed by “discontinuation of a medication,” and “increase in medication dose.” In the case of aspirin, it is usually not prescribed appropriately for secondary prevention of events, as recommended by certain guidelines such as the American Heart Association (AHA) Guidelines, and, for this reason, clinical pharmacists typically suggest the addition of aspirin to the patient’s regimen.^33^ For warfarin, CPIs would be related to dosing adjustments since this is highly common with this agent, and doses are susceptible to change based on the relative international normalized ratio (INR) readings. It is an important role of the clinical pharmacist in this setting to monitor INR levels and associate them with the relative warfarin dosing regimen, in addition to performing cardiovascular risk assessments for patients with prior cardiovascular events in order to recommend secondary prevention regimens.^26,33^ Notably, only one CPI suggested the addition of a serum level under this pharmacological class, and this also suggests the role of the clinical pharmacist in identifying the important serum levels required to monitor the effects of medications. Additionally, the second most commonly observed pharmacological class was that of the endocrine system and hormonal agents, where the medications that were frequently documented were insulin, glargine, and atorvastatin. The highly reported CPI under this class of medications was “increase in medication dose,” followed by “addition of another medication.” This finding was expected to be observed with a medication such as insulin glargine since the majority of diabetic patients who are hospitalized tend to show uncontrolled blood glucose control during admission due to stress and changes in diet and therapy regimens. In such cases, the role of the clinical pharmacists is significantly highlighted, where they would monitor the blood sugar levels on a daily basis and, therefore, implement relevant recommendations with regards to increasing the dose of insulin in order to achieve adequate control. Similarly, the documentation of atorvastatin in the CPIs relating to this pharmacological class could also be related to the primary and secondary prevention of cardiovascular events.

An additional significant result in this study was observed in the data pertaining to CPIs in the ED, in which the most frequently occurring CPI was related to the discontinuation of a medication, and this was seen as an inconsistency with previous studies that analyzed CPIs in the ED.^10,34^ Such studies reported that dose modification and inquiry of drug information were more abundantly observed. Furthermore, it is worth highlighting the crucial role of clinical pharmacists in conducting medication reconciliation at the time of admission and discharge, where this is the typical practice demonstrated in HH and all hospitals under HMC, and this is further supported by the fact that the most frequently occurring CPIs were attributed to the addition or discontinuation of medications as well as dosing adjustments. This is essential since it immensely aids in the therapeutic regimen planned for the patient during their hospital stay, and it is considered one of the most effective strategies to ensure that the appropriate medications are considered while avoiding any consequent patient harm and unnecessary monetary spending.^35^

This study involves several limitations. The retrospective nature of the study allows for a high chance of missed data pertaining to the CPIs as well as missing data about the general population. Consequently, studies that are prospective in nature are recommended. Also, the CPIs included in the study were not subject to content quality appraisal since it was assumed that the content verification would be completed by the clinical pharmacist placing the intervention and the prescribing physician who approved it.^36^ Furthermore, the female and male populations were uneven, noting that this may be attributable to the nature of the country’s population, where the most recent statistics report that males comprise more than 70% of Qatar’s population.^37^ Despite the presence of the mentioned limitations, this study reports considerably significant findings relating to the contribution of the clinical pharmacist in patient management in the cardiology setting, enhancing medication safety, and avoiding patient harm. This study also resulted in significant economic benefits in terms of cost savings and cost avoidance, as previously reported in our study,^38^ amounting to QAR1,595,948 (USD 438,447) per 3 months.

## Conclusion

This study highlighted a detailed overview of CPIs documented in the specialized HH, HMC, Qatar. Key findings revealed that the clinical pharmacists’ efforts in providing pharmaceutical care had posed a significant contribution to patient management, with presumed positive value for patient safety. Further focus may be beneficial when targeting the surveillance of the documented CPIs with regard to their content validity, precision, and quality. When it comes to medication use, multidisciplinary teams that include clinical pharmacists, working in harmony with other healthcare practitioners, would achieve better health outcomes for patients, particularly the prevention of the occurrence of DRPs.

## Source of Supporting and Funding

Authors have no conflict of interest to declare. This work was supported by Medical Research Center, Hamad Medical Corporation, Doha, Qatar [grant number (MRC-01-19-110)].

## Figures and Tables

**Table 1.** Characteristics of the study population.

		**Male [*n* = 158]**	**Female [*n* = 104]**	**Total [*n* = 262]**
Age (years), mean ± SD		56.0 ± 12.88	65.2 ± 12.14	60.9 ± 13.05
Weight (kg), mean ± SD		87.0 ± 25.94	84.6 ± 20.45	86.01 ± 23.89
	Arab	102 (38.93)	95 (36.26)	197 (75.19)
Ethnicity, *n* (%)	Asian (non-Arab)	54 (20.61)	7 (2.67)	61 (23.28)
	Other	2 (0.76)	2 (0.76)	4 (1.53)
	Inpatient (general cardiology)	127 (80.38)	82 (30.92)	209 (79.39)
Ward, *n* (%)	Inpatient (critical care)	10 (6.33)	6 (2.29)	16 (6.11)
	Emergency	16 (10.13)	15 (5.73)	31 (11.83)
	Heart failure clinic	5 (3.16)	1 (0.38)	6 (2.29)
	March 2018	241 (52.85)	215 (47.15)	456
Date range	July–August 2018	153	129	282
	January 2019			107

**Table 2.** Classification of interventions.

**Type of intervention**	**Count of type of change in resource, *n* (%)**
Addition of another medication	278 (32.90)
Discontinuation of a medication	196 (23.20)
Increase in medication dose	155 (18.34)
Decrease in medication dose	56 (6.63)
Switching to alternative medication	47 ( 5.56)
Addition of a laboratory test	46 (5.44)
Change in medication route	24 (2.84)
Others (patient education and medication reconciliation)	15 (1.78)
Decrease in medication frequency	13 (1.54)
Addition of a serum level	12 (1.42)
Decrease in medication duration	2 (0.24)
Increase in medication frequency	1 (0.12)
Grand total	845

**Table 3.** Types of interventions according to gender.

**Row labels**	**Female, *n***	**Male, *n***	**Total, *n***
Addition of a lab test	21	25	46
Addition of a serum level	3	9	12
Addition of another medication	133	145	278
Change in medication route	12	12	24
Decrease in medication dose	22	34	56
Decrease in medication duration	1	1	2
Decrease in medication frequency	6	7	13
Discontinuation of a medication	90	106	196
Increase in medication dose	82	73	155
Increase in medication frequency	1	0	1
Switching to alternative medication	26	21	47
Others (patient education and medicationreconciliation)	7	8	15
Total, *n*	404	441	845

**Table 4.** Number of interventions according to classes of medications.

**Class of medications**	**Interventions, *n* (%)**
Anti-infective agent	22 (2.61)
Blood derivatives and immunoglobulins	36 (4.26)
Cardiovascular drugs	393 (46.51)
Central nervous system agents	11 (1.32)
Endocrine system and hormonal agents	158 (18.7)
fluids and electrolytes	46 (5.45)
Gastrointestinal drugs	61 (7.22)
Multiple category	11 (1.31)
Musculoskeletal and joint disease drugs	6 (0.72)
Not medication	1 (0.12)
Vitamins and nutritional agents	17 (2.03)
Respiratory tract agent	11 (1.31)
Urinary tract disorders agents	3 (0.36)
Not reported	69 (8.17)

**Table 5.** Classes of medications according to age group.

**Class of medications**	**Adult, *n* (%)**	**Elderly, *n* (%)**	**Total, *n* (%)**
Anti-infective agent	12 (1.43)	10 (1.18)	22 (2.61)
Blood derivatives and immunoglobulins	19 (2.25)	17 (2.01)	36 (4.26)
Cardiovascular drugs	210 (24.85)	183 (21.66)	393 (46.51)
Central nervous system agents	6 (0.72)	5 (0.6)	11 (1.32)
Endocrine system and hormonal agents	86 (10.18)	72 (8.52)	158 (18.7)
Fluids and electrolytes	19 (2.25)	27 (3.2)	46 (5.45)
Gastrointestinal drugs	33 (3.91)	28 (3.31)	61 (7.22)
Multiple category	7 (0.83)	4 (0.48)	11 (1.31)
Musculoskeletal and joint disease drugs	4 (0.48)	2 (0.24)	6 (0.72)
Not medication	1 (0.12)	0	1 (0.12)
Not reported	39 (4.62)	30 (3.55)	69 (8.17)
Respiratory tract agent	4 (0.48)	7 (0.83)	11 (1.31)
Urinary tract disorders agents	1 (0.12)	2 (0.24)	3 (0.36)
Vitamins and nutritional agents	6 (0.72)	11 (1.31)	17 (2.03)

**Table 6.** Classes of medications according to ward.

**Row labels**	**Emergency**	**Heart failure clinic**	**Critical care**	**General cardiology**	**Grand total, number (%)**
Anti-infective agent	1	0	0	21	22 (2.61)
Blood derivatives and immunoglobulins	4	3	4	25	36 (4.26)
Cardiovascular drugs	45	11	20	317	393 (46.51)
Central nervous system agents	0	0	1	10	11 (1.32)
Endocrine system and hormonal agents	13	1	3	141	158 (18.7)
Fluids and electrolytes	3	1	2	40	46 (5.45)
Gastrointestinal drugs	8	0	3	50	61 (7.22)
Multiple category	3	0	1	7	11 (1.31)
Musculoskeletal and jointdisease drugs	0	0	1	5	6 (0.72)
Not medication	0	0	0	1	1 (0.12)
Not reported	9	2	5	53	69 (8.17)
Respiratory tract agent	2	0	0	9	11 (1.31)
Urinary tract disorders agents	0	0	0	3	3 (0.36)
Vitamins and nutritional agents	2	0	0	15	17 (2.03)
